# Effect of 808nm diode laser irradiation on root canal walls after smear layer removal: A scanning electron microscope study

**Published:** 2007-07-05

**Authors:** Masoud Parirokh, Mohammad Jafar Eghbal, Saeed Asgary, Jamileh Ghoddusi, Sally Stowe, Farshid Forghani, Arash Shahravan

**Affiliations:** 1 *Department of Endodontics, Dental School, Kerman University of Medical Sciences, Kerman, Iran*; 2 *Department of Endodontics, Dental Research Center, Shaheed Beheshti University of Medical Sciences, Tehran, Iran*; 3 *Department of Endodontics, Iranian Center for Endodontic Research, Dental School, Shaheed Beheshti University of Medical Sciences, Tehran, Iran.*; 4 *Department of Endodontics, Dental School, Mashhad University of Medical Sciences, Mashhad, Iran*; 5 *Facility Coordinator, Electron Microscope Unit, Australian National University, Canberra, Australia*

**Keywords:** Dentinal tubules, Diode laser, EDTA, Root canal, Smear layer

## Abstract

**Introduction:** This study was carried out to investigate the effect of 808nm diode laser irradiation on dentinal tubules of root canal wall.

**Materials and Methods:** Twelve single-rooted teeth were used. After cleaning and shaping with rotary instruments by the crown down technique, the smear layer was removed by alternating irrigation with EDTA and sodium hypochlorite. The teeth were then randomly divided into experimental and control groups of six teeth each. In experimental group, laser irradiation was activated inside the canal and the teeth of the other group served as controls. Kruskal-Wallis and Friedman tests were used for comparing occluded dentinal tubules in different part of the roots.

**Results:** Scanning electron microscopy showed that occluded dentinal tubules could be observed in all laser irradiated teeth; however, none of the control teeth showed occluded dentinal tubules. The Friedman test showed that in the laser irradiated group the best result was achieved in the apical third of the root canals compared with the middle (p<0.005) and cervical third (p<0.002). Dentinal tubules of the middle third were also significantly different from the cervical third as well (p<0.005).

**Conclusion:** Laser radiation after removing smear layer could successfully occlude dentinal tubules and the best results was achieved at the apical part of the canal.

## Introduction

Sanitation of the root canal system and the adjacent dentin has been always a requisite key for successful endodontic therapy (1).

Numerous effects have been reported after laser therapy by irradiation of the interior of the root canal, including removal of the smear layer, and morphological changes such as opening or closing dentinal tubules, fusion and recrystallization of the dentin (2-5). With regard to laser application in endodontics, laser systems such as neodymium: yttrium-aluminium-garnet (Nd:YAG) and carbon dioxide (CO2) lasers have proved effective in cleaning and disinfecting the root canal and lateral dentinal tubules (5).

Diode laser is a solid-state semiconductor laser that typically uses a combination of Gallium (Ga), Arsenide (Ar), and other elements such as Aluminium (Al) and Indium (In). Lasers may be designed to emit light over numerous wavelengths from violet to infrared, in continuous or pulsed modes. Diode lasers which have been introduced in endodontics generally use lines in the 800-980 nm range, which is poorly absorbed by water, and are operated in contact method using a flexible fiber optic delivery system (6).

Following development of the laser technique and device, the diode laser has gained increasing importance due to its compactness and low cost (1). Previous reports demonstrated the bactericidal effects of 810-nm wavelength (7-11). Safety in clinical use, the effects of diode laser on removal of smear layer and debris and degree of apical leakage in obturated teeth have been reported (12).

Research studies have shown that laser radiation can produce some alterations on the root canal wall. These alterations can change dentin permeability and hence decrease the penetration of fluids inside dentin tubules (1,3,5,9,13-15). It has been shown that micro-organisms invading a root canal can penetrate into dentinal tubules far distant from the root canal wall and therefore cannot be totally destroyed by irrigating solutions or disinfecting materials. One of the main reasons for laser treatment is the possibility of occluding dentinal tubules and thereby entrapping invading microorganisms.

However, to date, the potential application of 808-nm wavelength diode laser on root canal walls has seldom been addressed. Previous research study was focused on the ability of laser radiation to remove the smear layer (1); however, it has been shown that many new low cost rapid techniques could be employed for this purpose (16-17).

The aim of this study was to investigate the effect of 808nm diode laser on dentinal tubules after removing the smear layer.

## Materials and Methods

This study was approved by The Ethic Committee of Kerman University of Medical Sciences and The Human Ethic Committee of Australian National University (protocol No.2006/215). In this study, twelve extracted non-carious single-rooted maxillary human teeth with straight canals were used. The root surfaces of all specimens were cleaned before the experiment. Crowns were resected at the cementoenamel junction with a high-speed diamond bur under water irrigation and then discarded. The canals were cleaned and shaped by the crown down technique with K3 instruments (SybronEndo, Orange, CA, USA). The working length was visually determined 1 mm shorter than anatomic apical foramen. The root canals were irrigated with 3 mL of 5.25% NaOCl (Golrang, Tehran, Iran) between each file. After root canal preparation, the smear layer was removed by the application of 17% EDTA (Asiachimiteb, Tehran, Iran) for 1 minute followed by the irrigation with 5.25% NaOCl and finally the canal was irrigated with distilled water and dried with paper points (Aria, Tehran, Iran). The teeth were then randomly divided into experimental and control groups of 6 teeth each. A 808-nm wavelength GaAlAs diode laser device (Photolase, Milan, Italy) was used in this experiment. The maximum output was 2.5 W, and continuous mode was used. The delivery system consisted of flexible fiber that had 200µm diameter. Six teeth in group 1 were irradiated according to the manufacturer’s recommendation in the continuous mode at 2.5 W. During laser irradiation, each fiber tip was introduced into the root canal 1 mm shorter than working length and were kept in contact with the root canal walls, while moving in a circular motion at the cervical and central area and in a back and forth motion at the apical area of the root canal. The irradiation time was 10 second and this procedure was repeated for three times. Total irradiation time for each root canal was 30 seconds. The 6 teeth in control group were not irradiated.

All the teeth in both groups were longitudinally bisected by pliers after a single longitudinal groove was made on the labial and lingual root surfaces with a diamond disk. Each sample was put in an oven and allowed to dry at 50°C for 24 h. After drying, all samples were gold-coated using an Emitech (Ashford, UK) K550X sputter coater. A Cambridge Instruments Stereoscan 360 (Cambridge Instruments, Cambridge, UK) was used for imaging and observing basic morphology of dentinal tubules at an accelerating voltage of 15–20 kV.

Samples were viewed and evaluated at a magnification of x 800- 1500 and x 5000 for assessment of dentinal tubules’ patency. The tubules occluded on root canal walls were scored in a blind manner on a following four- grade scale independently by three examiners who were calibrated their responses on test samples to ensure consistency.

**Figure 1A:**
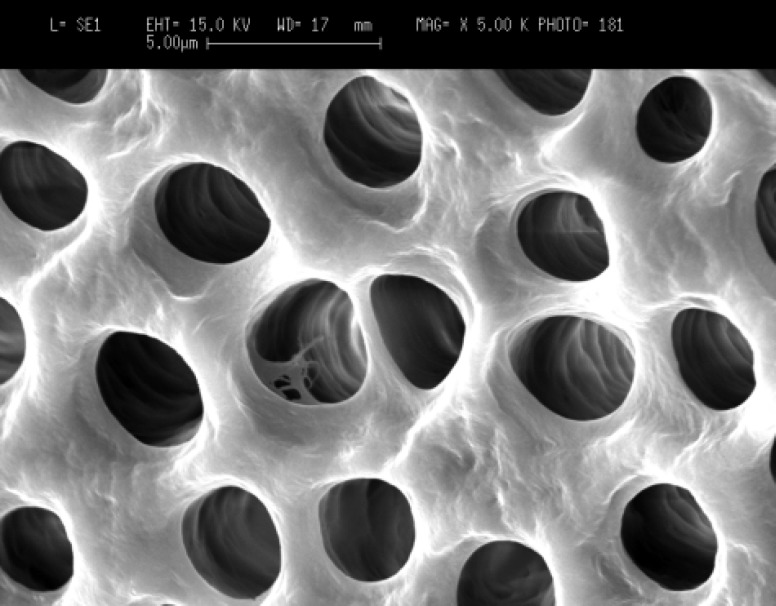
Patent dentinal tubules at the cervical third of the root canal in control group (original magnification x5000).

**Score 0**: absence of fused areas, open dentin tubules, **Score 1**: less than 50% of the dentin tubules occluded, **Score 2**: more than 50% of the dentin tubules occluded, **Score 3**: dentin fusion with rare open dentin tubules.

Both halves of each sample were evaluated in the apical, middle and coronal portions of the root canal walls. When discordant scores were reported on a sample by the examiners, the examination was repeated, and any further controversy was resolved by discussion.

Statistical analysis was performed using the Kruskal-Wallis and Friedman tests, and a value of *p *< 0.05 was considered significant.

## Results

Scanning electron microscopy (SEM) images showed that in the cervical third of the root canals of control samples the smear layer was successfully removed and patent dentinal tubules could be observed (Figure 1A). However, the dentinal tubules were occluded in the laser irradiated group in all apical, middle and cervical thirds of root canal wall. The Kruskal-Wallis test showed that there were significant differences between the laser irradiated and control groups in the apical, middle and cervical thirds (p<0.0001). The Friedman test showed that in the apical third of the irradiated root canals the amount of occluding dentinal tubules were significantly more than middle (p<0.005) (Figure 1B) and cervical areas (p<0.002) (Figures 2A, B, and C). Both control and irradiated root canal walls showed fewer dentinal tubules in the apical third (Figures 3A and B). In the apical third of the laser radiated samples most of the dentinal tubules were occluded, however, the control samples showed patent dentinal tubules. The amounts of occluded dentinal tubules in the middle third of the root canal wall were significantly more than in the cervical third (p<0.005). There was no sign of melting or recrystallization over the entrance of dentinal tubules in all apical, middle and cervical thirds of root canals which were irradiated by laser; however, it seems that the dentinal tubules were occluded by fusion of intertubular dentin. In the apical region more homogenous layer over dentinal tubules could be observed. Carbonization was not observed in any of the laser irradiated samples.

**Figure 1B:**
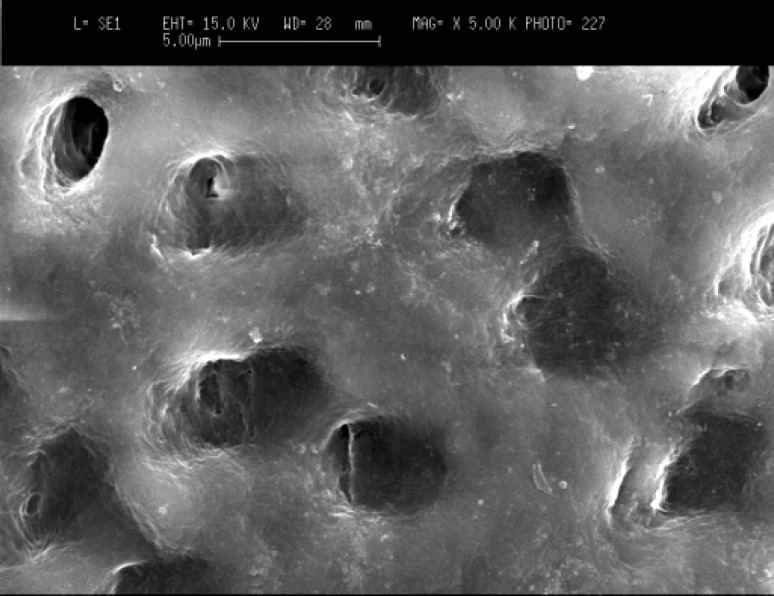
Occluded dentinal tubules at the cervical third of the root canal in laser irradiated group (original magnification x5000).

## Discussion

Following development of the laser technique and device, the diode laser has gained increasing importance due to its compactness and low cost (1).

Many different laser wavelengths have been investigated for use in the field of endodontics. Among them, the 808nm Nd:YAG laser is the most frequently used and accepted one (18). The relatively new diode laser is portable, compact and efficient for practical applications, such as to stop bleeding during surgical operation (1), to achieve disinfection and sterilization in root canal (7-11), and to relieve pain after treatment (6). Using a thin and flexible fiber means that the light can be delivered into narrow and curved root canals, which is of great value in endodontic applications. Wang *et al.* verified in their study that root canal preparation combined with 980nm wavelength laser irradiation was able to clean canal walls and open dentinal tubules, as well as reducing apical leakage (1). Researchers believe that as the penetration depth by their diode laser was lower than that of Nd:YAG laser (wavelength 1064nm), the risk of thermal side effects seems to be lower (1,6). In the present study a diode laser with 808nm wave length was used.

**Figure 2:**
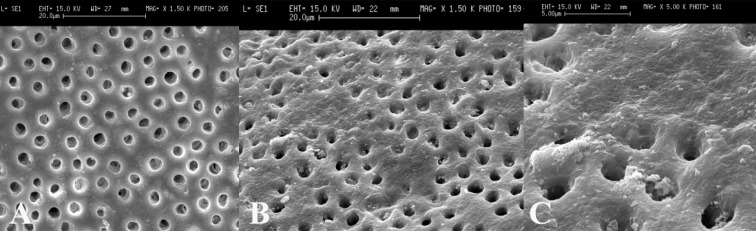
A) Patent dentinal tubules at the middle third of the root canal in control group (x1500), B)Partially occluded dentinal tubules at the middle third of the root canal in laser irradiated root canal (x1500), C) Higher magnification of B (x5000).

The action of lasers on tissues depends on the wavelength, power, pulse length, time of exposure, and type of tissue. (2,10,18)

In this study the laser was applied simulating clinical conditions, the result was a predominance of areas with obliterated tubules and compact surfaces, without the typical “melted” appearance characteristic of Nd:YAG laser used in previous research studies (4,18). The morphology of root canals also influences the laser performance in dentinal wall.

In the present study, using diode laser showed that the major morphologic changes has occurred at the apical third of the root canal, however, Anic *et al.* (14) as well as Camargo *et al.* (18), using Nd:YAG laser, verified that the cervical and apical third of the specimens submitted to the laser showed larger and smaller morphological alterations, respectively. Variation between the power and wavelength of the lasers may be responsible for this difference. Moritz *et al.* (9), as well as the present study showed both fusion and closure of dentinal tubules. However, Wang *et al*. could not observe dentin fusion or closure of dentinal tubules. They attributed those differences to the variation of wavelength, mode of laser irradiation, power, and exposure time or fiber diameter (1).

In this study, the most dentinal tubule closure was achieved at the apical third of the root canal wall. This can be attributed to the narrower canal in the apical third (i.e., the narrower the root canal, the closer contact the laser tip with canal walls), thus more laser radiation could be absorbed by the root canal walls. Wang *et al*. showed that the apical third was much cleaner than both cervical and middle third of the root canal. However, they had not removed smear layer before laser submission (1).

Previous research studies have emphasized that the fiber should be kept in constant motion in the root canal during irradiation to avoid rise in temperature on the root surface (1,6). For this reason, in this study quick motions were employed.

When the laser light is exposed to the canal walls, part of the beam is absorbed by the water in the dentin, thus heating the irradiated area to very high temperatures within a short period of time. The amount of energy that is absorbed by the dentin can cause vaporization or fusion as the heat spreads through the tissue. In endodontic therapy, this fact results in the opening or closing of the dentinal tubules depending on the rate of absorption.

Lasers with different wavelengths have various effects when applied for the same tissue, because the interaction of light with the biological tissue depends on the absorption of that particular radiation. The interaction between the tissue and the laser is also dependent on parameters such as average power, frequency, and exposure time (19). Thus, by comparing the effects on the dentin wall of the radicular canal after Nd:YAG and Er:YAG laser irradiation, Tanji *et al. *(20) concluded that the Er:YAG laser promoted ablation and opened the dentinal tubules, while the Nd:YAG laser closed them by promoting coalition and recrystallization of the dentin.

**Figure 3A:**
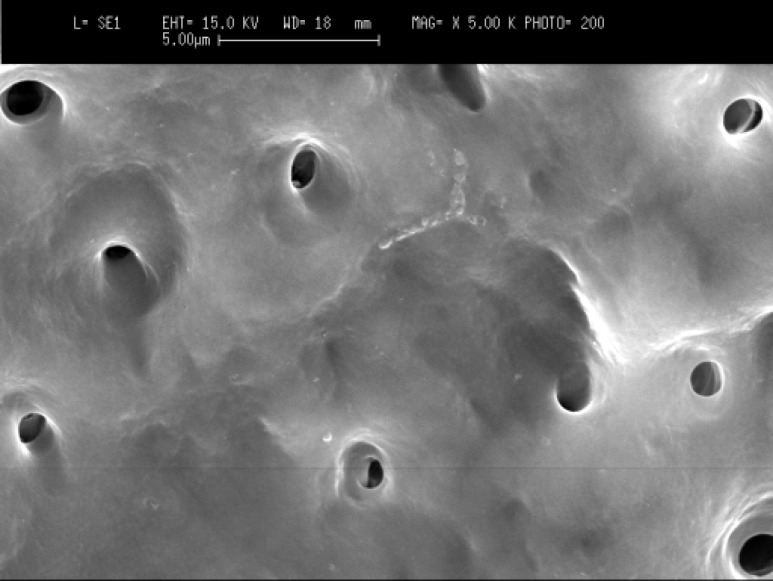
Patent dentinal tubules at the apical third of the root canal in control group (original magnification x5000).

Folwaczny *et al.* (21) suggested that use of the laser in the root canal walls leads to a reduction in the number of opened tubules, promoting a decrease of apical permeability, and this reduction in the permeability of the dentin walls can improve the sealing of root canal filling. During irradiation, the root canal debris was vaporized with ablation of the canal wall resulting in marked craters, carbonization, glazed surface and melted dentinal tubules, depending on the laser energy and irradiation parameters. Removing the debris, reducing the diameter and number of openings of dentinal tubules and obtaining a glazed canal wall conceivably decreased the dentin permeability in the apical area of the canal.

Many previous studies focused on the utility of laser beams for debris removal and promotion of better adhesion between root canal sealer and root canal wall (1,4,18). However, producing patent dentinal tubules may always present a risk of infected root canals because it has been shown that micro-organisms can penetrate more than 2000 microns into dentinal tubules (22).

**Figure 3B:**
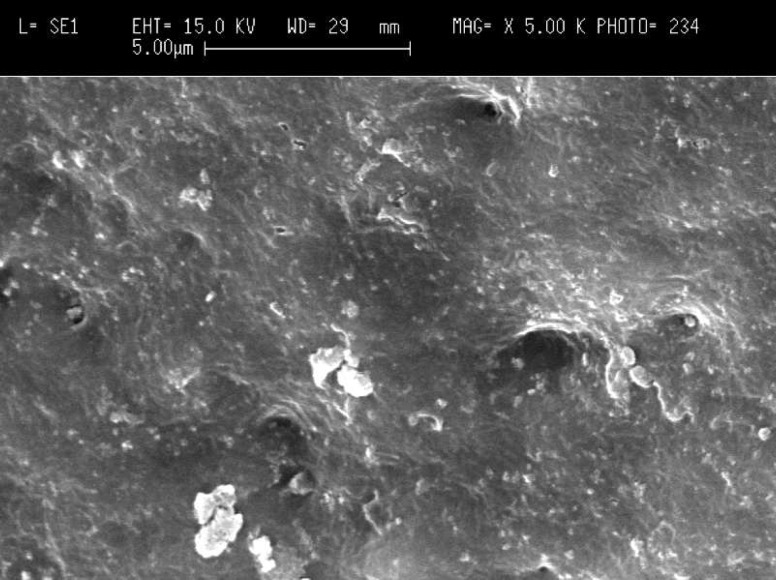
Occluded dentinal tubules at the apical third of the root canal in laser irradiated root canal (original magnification x5000).

Therefore, if the apical seal is compromised and root canal sealer washes out by apical percolation patent dentinal tubules would be pathways for micro-organisms. However, if the smear layer could be removed with other methods such as using EDTA and sodium hypochlorite, most laser radiation will be absorbed by root canal walls and produce occluded dentinal tubules which will decrease the risk of reinfection. In this study, laser radiation was employed after smear layer removal, and occlusion of dentinal tubules particularly in the apical third area was observed.

In this study, for removing the smear layer after root canal instrumentation the canal was irrigated by EDTA for 1 minute followed by 3 mL of 5.25% NaOCl. Crumpton *et al.* (17) in their recently published study have shown that efficient removal of the smear layer was accomplished with a final rinse of the same amount of EDTA and NaOCl. Our SEM observation confirmed their results as most dentinal tubules of control samples were patent and free of smear layer.

## Conclusion

The results of this study have shown that laser radiation after removing smear layer could successfully occlude dentinal tubules and the best results was achieved at the apical part of the canal. Although diode laser irradiation has beneficial effects on morphology of root canal walls, further research studies for clinical application should be performed.
